# Evaluation of Genetic Diversity in Quill Mites of the Genus *Syringophiloidus* Kethley, 1970 (Prostigmata: Syringophilidae) with Six New-to-Science Species

**DOI:** 10.3390/ani13243877

**Published:** 2023-12-16

**Authors:** Eliza Glowska, Izabella Laniecka, Kamila Ostrowska, Christina A. Gebhard, Julia Olechnowicz, Miroslawa Dabert

**Affiliations:** 1 Department of Animal Morphology, Faculty of Biology, Adam Mickiewicz University in Poznań, Uniwersytetu Poznańskiego 6, 61-614 Poznan, Poland; izabella.laniecka@gmail.com (I.L.); kamromanowska@gmail.com (K.O.); 2 Division of Birds, Smithsonian Institution, MRC 116, P.O. Box 37012, Washington, DC 20013-7012, USA; gebhardc@si.edu; 3 Molecular Biology Techniques Laboratory, Faculty of Biology, Adam Mickiewicz University in Poznań, Uniwersytetu Poznańskiego 6, 61-614 Poznan, Poland; julia.olechnowicz@amu.edu.pl (J.O.); miroslawa.dabert@amu.edu.pl (M.D.)

**Keywords:** quill mites, bird parasites, molecular taxonomy, DNA barcoding, COI

## Abstract

**Simple Summary:**

Morphology and barcode data were used to estimate the diversity and genetic variability of fourteen putative species of the genus *Syringophiloidus* Kethley, 1970. In most cases, both sources of information were consistent. The only exception was *S. amazilia* Skoracki, 2017, which according to our results is most likely a population of *S. stawarczyki* Skoracki, 2004, and probably should be treated as its junior synonym. The further findings of our study are six new-to-science species described herein. We indicate that both the host phylogeny and distribution can drive the evolution of quill mites. Our results increase the knowledge of quill mite diversity and provide some premises to formulate and further test evolutionary, ecological, and epidemiological inquiries.

**Abstract:**

Quill mites (Acariformes: Syringophilidae) are poorly explored bird parasites. *Syringophiloidus* Kethley, 1970, is the most specious and widespread genus in this family. It is believed to contain mono-, steno- and poly-xenous parasites and thus seems to be an exemplary for studies on biodiversity and host associations. In this work, we applied the DNA barcode marker (mitochondrial cytochrome c oxidase subunit I gene fragment, COI) to analyze the species composition and host specificity of representatives of fifteen *Syringophiloidus* populations parasitizing fifteen bird species. The neighbor joining analyses distinguished thirteen monophyletic lineages, almost completely corresponding to seven previously known species recognized based on morphological features, and six new-to-science species. The only exception is *S. amazilia* Skoracki, 2017, which is most likely conspecific with *Syringophiloidus stawarczyki* Skoracki, 2004. The intraspecific distances of all species were not higher than 0.9%, whilst the interspecific diversity ranged from 5.9% to 19.2% and 6.3–22.4%, inferred as the distances *p* and K2P, respectively. Although all putative species (except *S. amazilia*) are highly supported, the relationships between them have not been fully resolved and only faintly indicate that both host phylogeny and distributions influence the phylogenetic structure of quill mite taxa.

## 1. Introduction

Quill mites (Acariformes: Syringophilidae) are widespread permanent bird ectoparasites. To date, 417 species have been described [1,2], although their actual number is estimated to be several times higher, probably reaching up to 5000 species [3]. Although the knowledge about syringophilid diversity and host associations has been growing recently [4,5,6], they remain one of the least understood bird parasites. This is due to their small body size, poorly accessible habitats (bird’s feather quill), and low prevalence [7]. Further difficulties are caused by weakly informative morphology and relatively few diagnostic characters [8,9]. Moreover, the vast majority of species were described only on the basis of female features, and the consequence is that males, nymphs, and larvae are virtually unidentifiable. To overcome the limitations of morphology, molecular methods have recently come into use in mite taxonomic studies [9,10,11]. DNA barcoding is an approach employing a short fragment of the mitochondrial cytochrome c oxidase subunit I (COI) sequence. It is commonly used as an effective marker in the process of species identification in many groups of animals (Hebert 2003, 2004) [12,13], including quill mites [14]. Although only a very small fraction of those parasites have been barcoded so far, this approach has proven reliable in such systematic inquiries as female dimorphism or phenotypic plasticity [8,9]. It has also been successfully used for the estimation of host spectrum [15] and cryptic species detection [16].

Precise and unambiguous species diagnosis is crucial for any other research, including that on quill mites’ parasitological and epidemiological importance. This is particularly important in the context of recent reports that mites are the host of unique phylogenetic lineages of bacteria of the genera *Wolbachia* and *Spiroplasma*. In addition, the presence of *Bartonella* and *Brucella* taxa has been detected in syringophilids, which makes them potentially important in the process of circulation of pathogens among birds [17].

The *Syringophiloidus* Kethley, 1970, is the most specious and widely distributed genus of quill mites with 48 known species widespread around the world. This taxon has been recorded from 80 avian host species, belonging to 29 families and five orders [1,2]. Since the species of this genus are known to have various (mono-, steno- and poly-xenous) associations with hosts, they seem to be a representative material for research on diversity and host associations.

In this paper, we supplement the morphology with DNA barcode coverage to evaluate the species composition and host specificity of representatives of fifteen *Syringophiloidus* populations parasitizing fifteen selected bird species.

## 2. Materials and Methods

### 2.1. Animal Material and Morphological Analysis

Mite material used in the study (Table 1) was acquired from several sources: (i) the collection of feathers deposited in the Smithsonian Institution, National Museum of Natural History, Department of Vertebrate Zoology, Division of Birds, Washington, DC, USA (USNM) (September 2014), and bird specimens originally collected in Gabon (2009), Namibia (2009), and Peru (2009); (ii) the Biocenter Grindel and Zoological Museum (University of Hamburg), and bird specimens originally collected in Tanzania; (iii) mite samples collected in Mexico (field no. SVM 08-0506-1/4) (2008) and Brazil (2010); (iv) mites obtained from dead birds (due to probable collisions with the window glass) found at the AMU campus, Poznań, Poland (2009).

Drawings were made with an Olympus BH2/BX41/BX53 microscopes with differential interference contrast (DIC) optics and a camera lucida. All measurements are in micrometers (µm). Idiosomal setation follows that of [18] with modifications adapted for Prostigmata by [19]. The nomenclature of leg chaetotaxy follows that proposed by [20]. The application of this chaetotaxy to Syringophilidae was recently provided by [21] with a few changes by [22]. Latin and common names of the birds follow [23].

*Material depositories and abbreviations*: AMU—Adam Mickiewicz University, Poznań, Poland; USNM—Smithsonian Institution, National Museum of Natural History, Washington, DC, USA. The voucher slides and corresponding DNA samples are deposited in the collection of the AMU and USNM under the identification numbers indicated below. The sequences are deposited in GenBank under accession nos. specified in Table 1.

### 2.2. Molecular Data and Analysis

Total genomic DNA was extracted from single specimens using DNeasy Blood & Tissue Kit (Qiagen GmbH, Hilden, Germany) as described by [24]. The COI gene fragment was amplified via PCR with degenerate primers: Aseq01F (GGAACRATATAYTTTATTTTTAGA) and Aseq03R (GGATCTCCWCCTCCWGATGGATT) [9]. PCR amplifications were carried out in 10 µL reaction volumes containing 5 µL of Type-it Microsatellite Kit (Qiagen), 0.5 µM of each primer, and 4 µL of DNA template using a thermocycling profile of one cycle of 5 min at 95 °C followed by 35 steps of 30 s at 95 °C, 1 min at 50 °C, and 1 min at 72 °C, with a final step of 5 min at 72 °C. After amplification, PCR products were diluted two-fold with water, and 5 µL of the sample was analyzed via electrophoresis on 1.0% agarose gel. Samples containing visible bands were purified with thermosensitive Exonuclease I and FastAP Alkaline Phosphatase (Fermentas, Thermo Scientific, Waltham, MA, USA). The amplicons (585 bp) were sequenced in one direction using the Aseq01F primer. Sequencing was performed with BigDye Terminator v3.1 on ABI Prism 3130XL Analyzer (Applied Biosystems, Foster City, CA, USA). Sequence chromatograms were checked for accuracy and edited using Geneious R11 (Biomatters Ltd., Auckland, New Zealand).

Phylogenetic associations between the studied taxa were estimated with the neighbor joining (NJ) method implemented in MEGA7 [25]. Support for the recovered trees was evaluated with 1000 (NJ) non-parametric bootstrap replicates [26]. Pairwise distances between nucleotide COI sequences were calculated using Kimura’s two-parameter (K2P) and distance *p* models [27] for all codon positions with MEGA7. *Stibarokris phoeniconaias* Skoracki and OConnor, 2010, was chosen as an outgroup to root the tree. Tree visualizations were prepared using tree editing tools in MEGA7 and Figtree v.1.4.2—[28] (http://tree.bio.ed.ac.uk/) URL (accessed on 15 October 2023).

## 3. Results

### 3.1. Systematics

Family Syringophilidae Lavoipierre
Subfamily Syringophilinae Lavoipierre
Genus *Syringophiloidus* Kethley

### 3.2. Molecular Analysis

We provided DNA barcode coverage for the representation of fifteen populations of *Syringophiloidus* ssp. recorded from fifteen bird species. The COI alignment was 552 bp long and comprised 43 sequences of *Syringophiloidus* mites (ingroup) and one sequence of *Stibarokris phoeniconaias* Skoracki & OConnor, 2010 (outgroup). The number of sequences obtained from each mite population varied from 1 to 10. The alignment contained 242 variable sites, 196 of which were parsimony informative.

**Figure 1:**
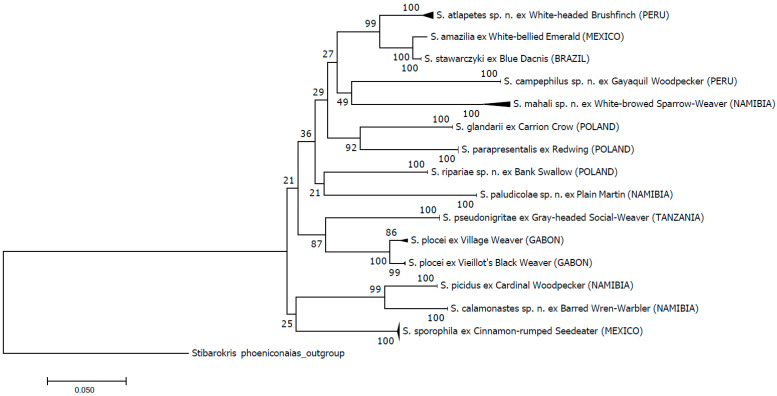
Neighbor joining phylogenetic tree of the *Syringophiloidus* species based on the K2P model. The tree was constructed in Mega v.7. and rooted by *Stibarokris phoeniconaias*.

**Figure A1:**
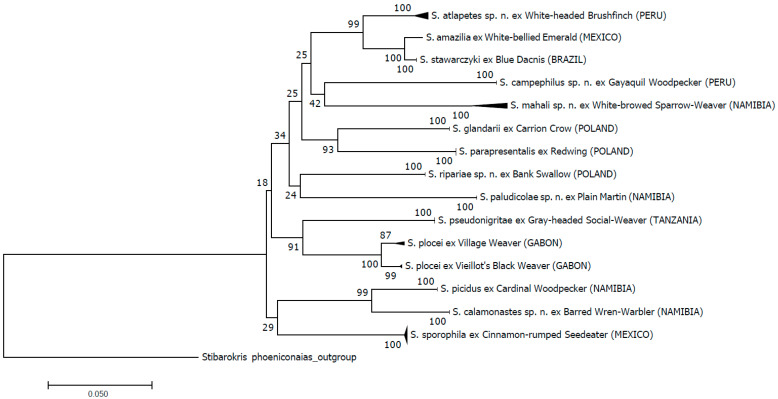
Neighbor-joining phylogenetic tree of the *Syringophiloidus* species based on the *distance p* model. The tree was constructed in Mega v.7. and rooted by *Stibarokris phoeniconaias*.

The neighbor joining phylogenetic analyses (K2P and distance *p*) distinguished thirteen monophyletic lineages, among which seven lineages exactly correspond to seven previously known and species that are morphologically distinguished here. The only exception in the obtained pattern is presented by *S. amazilia*, which is very close to that of *S. stawarczyki* and most likely represents a population or subspecies of this species (Figure 1 and Figure A1).

This assumption is also supported by the genetic distance between the two populations (1.4 and 1.5% of *distance p* and K2P) (Table 2), which is lower than that between bihost *S. plocei* populations (2.1%) and comparable to the previously reported intraspecific values within other quill mites [8]. Although all putative species are highly supported with bootstrap values (100%) and, as they predictably delineate the morphospecies, the relationships between them have not been fully resolved and only weakly suggest various evolutionary scenarios.

The genetic distances were compared at intra- and inter-specific levels according to both the *distance p* and the K2P model. The integrity and separateness of particular taxa were proven for almost all populations resulting in the recognition of seven previously known species and six species new to science. The intraspecific distances of all species were not higher than 0.9%, whilst the interspecific diversity ranged from 5.9% to 19.2% and 6.3–22.4% for genetic distances *p* and K2P, respectively (Table 2).

### 3.3. Morphological Systematics

#### 3.3.1. Descriptions

##### *Syringophiloidus atlapetes* sp. n. (Figure 2 and Figure 3)

**Figure 2:**
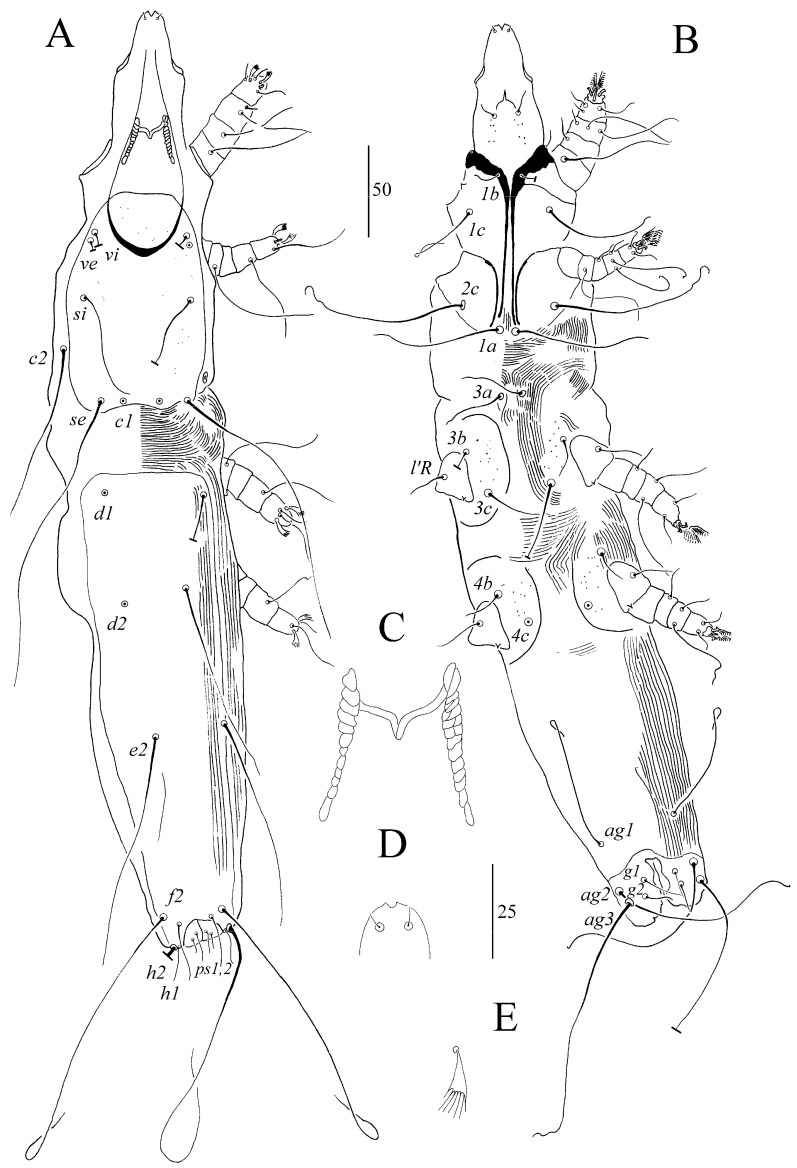
(**A**–**E**). *Syringophiloidus atlapetes* sp. n., female: (**A**) dorsal view, (**B**) ventral view, (**C**) peritremes, (**D**) hypostomal apex, and (**E**) fan-like setae *p’* of leg III. Scale bars: (**A**,**B**) = 50 µm; (**C**–**E**) = 25 µm.

For females (holotype and three paratypes; range in parentheses) (Figure 2A–E), the total body length is 605 (600–620). For *Gnathosoma*, the infracapitulum is punctate. Each medial and lateral branch of peritremes has 2–3 and 9–11 chambers, respectively (Figure 2C). The stylophore is punctate and has a body length of 165 (150–155). For *Idiosoma,* the propodonotal shield is rounded anteriorly and sparsely punctate on the entire surface. The length ratio of setae *vi*:*ve*:*si* is 1:1:3.3–4.6. The hysteronotal shield is clearly visible and punctate in anterior and posterior parts. The pygidial shield is punctate and distinctly sclerotized in the area bearing bases of setae *f1* and *f2*. Setae *f1* and *h1* are subequal in length. The length ratio of setae *ag1*:*ag2*:*ag3* is 1.1:1:1.2. For *Legs*, Coxal fields I–IV are sparsely punctate. Setae *3c* is 3.4–3.6 times longer than *3b*. Fan-like setae *p’* and *p”* of legs III–IV have seven tines (Figure 2E). Setae *tc”* is 1.3–1.6 times longer than *tc’*. Lengths of setae are as follows: *vi* 15 (20); *ve* 15 (20); *si* 70 (65–75); *c2* 160 (185); *se* 205 (195–225); *c1* 215; *d2* 175 (165); *d1* 145 (145); *e2* 125 (140–170); *f1* 20 (25); *f2* 180 (205); *h1* 25 (20); *h2* 295 (285); *ag1* 125; *ag2* 115 (125–135); *ag3* 160; *g1*, *g2* 25 (25); *ps1* 12 (12); *ps2* 17 (17), *tc’* (30–40); *tc”* (50); *l’RIII* 35 (40–45); *l’RIV* (25); *3b* 25 (15); *3c* 85 (55–75); *4b* 20 (20); *4c* 85 (55).

**Figure 3:**
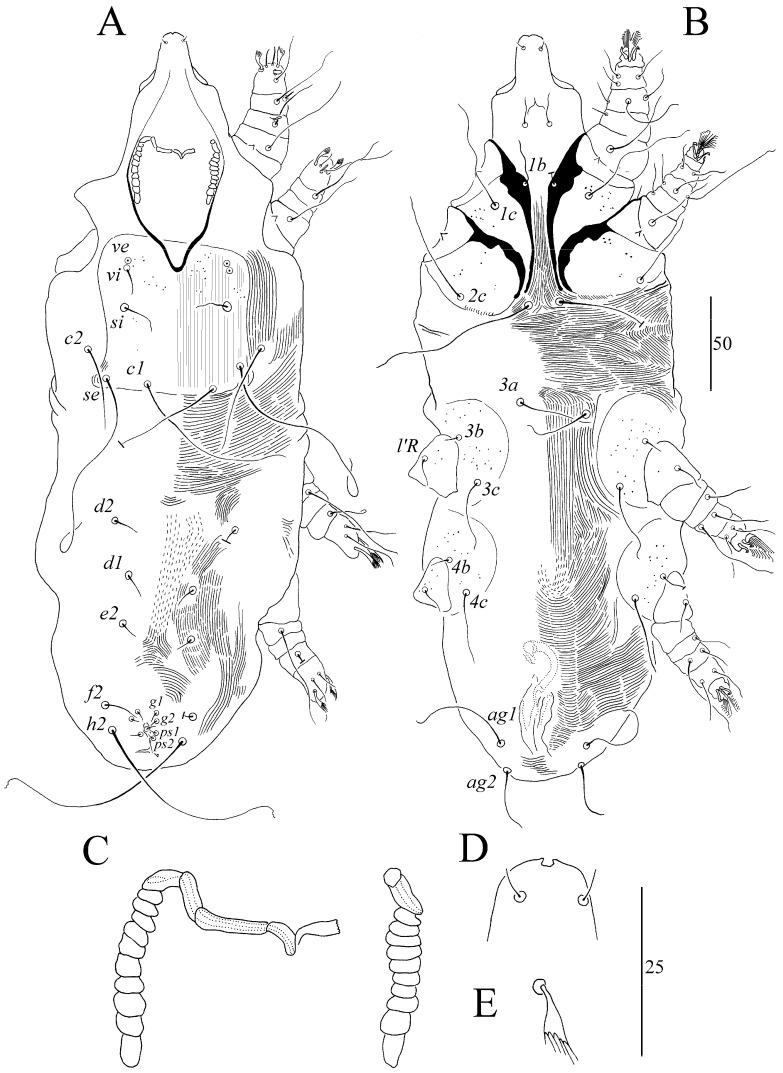
(**A**–**E**). *Syringophiloidus atlapetes* sp. n., male: (**A**) dorsal view, (**B**) ventral view, (**C**) peritremes, (**D**) hypostomal apex, and (**E**) fan-like setae *p’* of leg III. Scale bars: (**A**,**B**) = 50 µm; (**C**–**E**) = 25 µm.

For males (paratype) (Figure 3A–E), the total body length is 400. For Gnathosoma, the infracapitulum is apunctate. The stylophore is apunctate and 130 long. Each medial branch of peritremes has four chambers, and each lateral branch has 10 chambers (Figure 3C). For Idiosoma, the propodonotal shield is weakly sclerotized, bearing bases of setae *vi*, *ve*, *si*, *se*, and *c1*, and sparsely punctate near bases of setae *vi*, *ve* and *si*. Striation is clearly visible on the entire surface. The length ratio of setae *ve*:*si* is 1:1. The hysteronotal shield is weakly sclerotized, and the striae are visible, not fused to a pygidial shield, and apunctate. Setae *d1*, *d2*, and *e2* are subequal in length. The pygidial is shield small, restricted to bases of setae *f2* and *h2*, and to the genito-anal region or only to the genito-anal region; it is apunctate. Genital setae *g1* is situated anterior to the level of setae *g2*, and both pairs are subequal in length. Pseudanal setae *ps1* and *ps2* are subequal in length. Length ratios of setae *ag1*:*ag2* and *f2*:*h2* are 1.3:1 and 1:11.5, respectively. Coxal fields I–IV are punctate. Setae *3c* is four times longer than *3b*. For legs, fan-like setae *p’* and *p”* of legs III and IV have 6 tines (Figure 3E). The length ratio of setae *tc’III*–*IV*:*tc”III*–*IV* is 1:1.7. The lengths of setae are as follows: *ve* 15, *si* 15, *se* 100, *c1* 100, *c2* 55, *d1* 13, *d2* 13, *e2* 13, *f2* 10, *h2* 115, *ag1* 45, *ag2* 35, *3b* 10, *3c* 40, *l’RIII* 13, *l’RIV 15 tc’III–IV* 15, and *tc”III–IV* 25.

Host and Distribution

Birds of the family Passerellidae: the white-headed brushfinch, *Atlapetes albiceps* (Taczanowski) from Peru.

Type Material

The type material included a female holotype, and seven female and one male paratypes from the quill of the white-headed brushfinch, *Atlapetes albiceps* (Taczanowski) (Passeriformes: Passerellidae), PERU, Tumbes, Parque Nacional Cerros de Amotape, El Platano, 4 07 46 S, 80 37 13 W, 11, 13 July 2009, coll. Milensky, C. M, (USNM 643973). Mites were sampled by Glowska E.; the vouchers and DNA codes are as follows: EG974–977. DNA barcode GenBank accession numbers as specified in Table 1.

Type Material Deposition

The female holotype (USNMENT acc. number: USNMENT01967000) and four paratypes (three females and one male) (USNMENT01967001–USNMENT01967004) are deposited in the USNM, and four female paratypes are deposited in the AMU (EG23-0628-003.01-04).

Differential Diagnosis

*Syringophiloidus atlapetes* sp. n. is morphologically most similar to *S. stawarczyki* Skoracki, 2004, described from the golden-rumped euphonia *Euphonia cyanocephala* (Vieillot) (Passeriformes: Fringillidae) and additionally recorded from the white-lined tanager, *Tachyphonus rufus* (Boddaert), and the blue dacnis, *Dacnis cayana* (L.) (Passeriformes: Thraupidae) [29,30]. Females of both species have a similar number of peritremal chambers, punctate dorsal shields, and Coxal fields I–IV, as well as similar or nearly coinciding lengths of most setae. Females of *S. atlapetes* sp. n. differ from those of *S. stawarczyki* in terms of a stylophore length of 150–165 (vs. that of 170–195 in *S. stawarczyki*), lengths of the setae *se* of 200–225 (vs. 165–170) and *d2* 165–175 (vs. 115–125), and the sparse punctation of the dorsal and Coxal shields (vs. dense punctation). The genetic distance between both species is 6.3% of K2P and 5.9% of distance *p*. *S. atlapetes* sp. n. is also very similar to *S. coccothraustes* Skoracki, 2011, described from the hawfinch *Coccothraustes coccothraustes* (L.) (Passeriformes: Fringillidae) [22]. Females of both species have a similar number of peritremal chambers, punctate dorsal shields, and Coxal fields I-IV, as well as coinciding lengths of most setae. Females of *S. atlapetes* sp. n. differ from those of *S. coccothraustes* in terms of the lengths of setae *vi* of 15–20 (vs. 25–35), *ve* of 15–20 (vs. 25–35), *h2* of 285–295 (vs. 305–330), *g1* and *g2* of 25 (vs. 35–40), and *tc”* of 50 (vs. 70).

Etymology

The name is taken from the generic name of the host and is a noun in apposition.

##### *Syringophiloidus calamonastes* sp. n. (Figure 4A–E)

**Figure 4:**
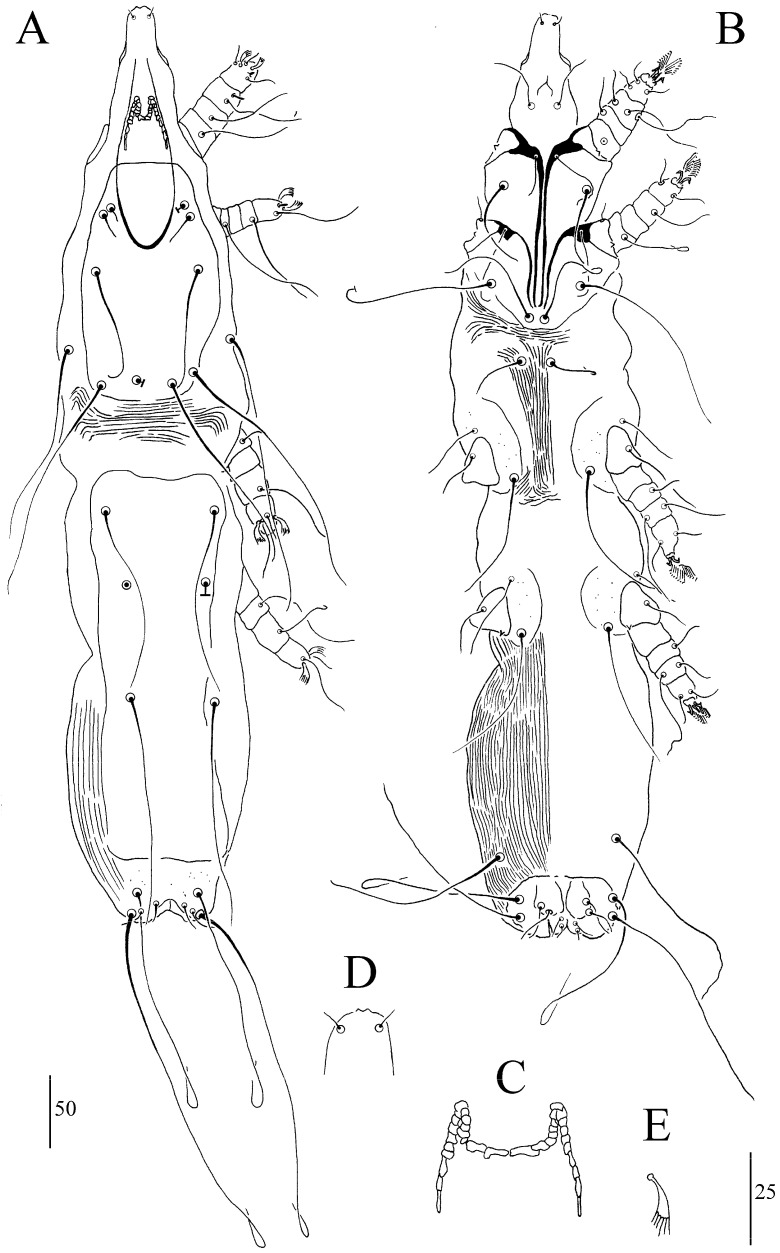
(**A**–**E**). *Syringophiloidus calamonastes* sp. n., female: (**A**)—dorsal view, (**B**)—ventral view, (**C**)—peritremes, (**D**)—hypostomal apex, (**E**)—fan-like setae *p’* of leg III. Scale bars: (**A**,**B**) = 50 µm; (**C**–**E**) = 25 µm.

Female (holotype and 7 paratypes; range in parentheses). Total body length 715 (645). *Gnathosoma*. Infracapitulum apunctate. Each medial and lateral branch of peritremes with 6–8 and 8–10 chambers, respectively (Figure 4D). Stylophore apunctate, 155 (155) long. *Idiosoma.* Propodonotal shield rounded anteriorly and apunctate. Length ratio of setae *vi*:*ve*:*si* 1:1.4–2.3:2.2–3.5. Hysteronotal shield strongly sclerotized and apunctate, fused to pygidial shield. Pygidial shield punctate, distinctly sclerotized in the area bearing bases of setae *f1* and *f2*. Setae *h1* 1.2–1.5 longer than *f1*. Length ratio of setae *ag1*:*ag2*:*ag3* 1–1.2:1:1.3–1.6. Genital plate present, bearing bases of setae *ag2* and *ag3*. *Legs.* Coxal fields I–IV apunctate. Setae *3c* 2.6–3.2 times longer than *3b*. Fan-like setae *p’* and *p”* of legs III–IV with 6–7 tines (Figure 4E). Setae *tc”* 2–2.8 times longer than *tc’*. *Lengths of setae*: *vi* 25 (20–25); *ve* 35 (35–45); *si* 55 (55–70); *c2* 155 (130–170); *se* 170 (165–205); *c1* 200 (170–180); *d2* 145 (130–180); *d1* 105 (105–145); *e2* 155 (145–155); *f1* 20 (25); *f2* 145 (160); *h1* 30 (30–35); *h2* 340 (260–295); *ag1* 125 (115–130); *ag2* 110 (95–130); *ag3* 160 (150–165); *g1*, *g2* 30 (30–35); *ps1,2* 15 (15–20); *tc’* 20 (20–25); *tc”* 55 (45–60); *l’RIII* 35 (25); *l’RIV* 35 (35); *3b* 25 (20–25); *3c* 65 (65–75); *4b* 35 (25–30); *4c* 60 (70–80).

Male: not found.

Host and Distribution

Birds of the family Cisticolidae: southern barred warbler *Calamonastes fasciolatus* (Smith) from Namibia.

Type Material

Female holotype and 10 female paratypes from the quill of the Southern Barred Warbler *Calamonastes fasciolatus* (Smith) (Passeriformes: Cisticolidae), NAMIBIA, Erongo, Tubusis, 21 39 46 S, 15 23 44 E, 4 Sep 2009, bird specimen coll. Gebhard, C. A. (USNM 642616), mites sampled by Glowska E. (15 Sep 2013); vouchers and DNA codes: KR043, KR045; DNA barcode GenBank accession numbers as specified in Table 1.

Type Material Deposition

Female holotype (USNMENT acc. number: USNMENT01967005) and 5 paratypes (USNMENT01967006–USNMENT01967010) are deposited in the USNM and 5 female paratypes in the AMU (EG23-0628-001.01–05).

Differential Diagnosis

*Syringophiloidus calamonastes* sp. n. is morphologically most similar to *S. picidus* Skoracki, Klimovičová, Muchai and Hromada, 2014 described from the cardinal woodpecker *Dendropicos fuscescens* (Vieillot) (Piciformes: Picidae) and recorded in Kenya, Tanzania and Uganda [31]. Females of both species have a similar number of peritremal chambers, all propodonotal setae serrated, fused, posteriorly punctate hystero-pygidial shield, and pseudanal setae *ps1* and *ps2* subequal in length. Females of *S. calamonastes* sp. n. differ from *S. picidus* by apunctate infracapitulum, propodonotal shield and coxal fields (vs. punctate in *S. picidus*), length ratio of setae *vi*:*ve*:*si* 1:1.4–2.3:2.2–3.5 (vs. 1:1.2–1.3:1.7–2.3) and equal genital setae (vs. *g1* 1.2 longer than *g2*). Our molecular analysis revealed that both species differ by 7.3% of K2P (and 6.9% of distance *p*). *S. calamonastes* sp. n. is also very similar to *S. minor* (Berlese, 1887) described from the house sparrow *Passer domesticus* (L.) (Passeriformes: Passeridae) from Europe and additionally recorded from several species and localities around the world [22]. Females of both species have a similar number of peritremal chambers, fused hysteronotal and pygidial shields, fan-like setae *p’* and *p”* of legs III–IV with 6–7 tines and same lengths of most setae. Females of *S. calamonastes* sp. n. differ from *S. minor* by apunctate infracapitulum and propodonotal shield (vs. punctate in *S. minor*) and the lengths of setae *se* 165–205 (vs. 150–160), *e2* 145–155 (vs. 105–135), *tc’* 20–25 (vs. 40), and *tc”* 45–60 (vs. 75–80).

Etymology

The name is taken from the generic name of the host and is a noun in apposition.

##### *Syringophiloidus campephilus* sp. n. (Figure 5A–E)

**Figure 5:**
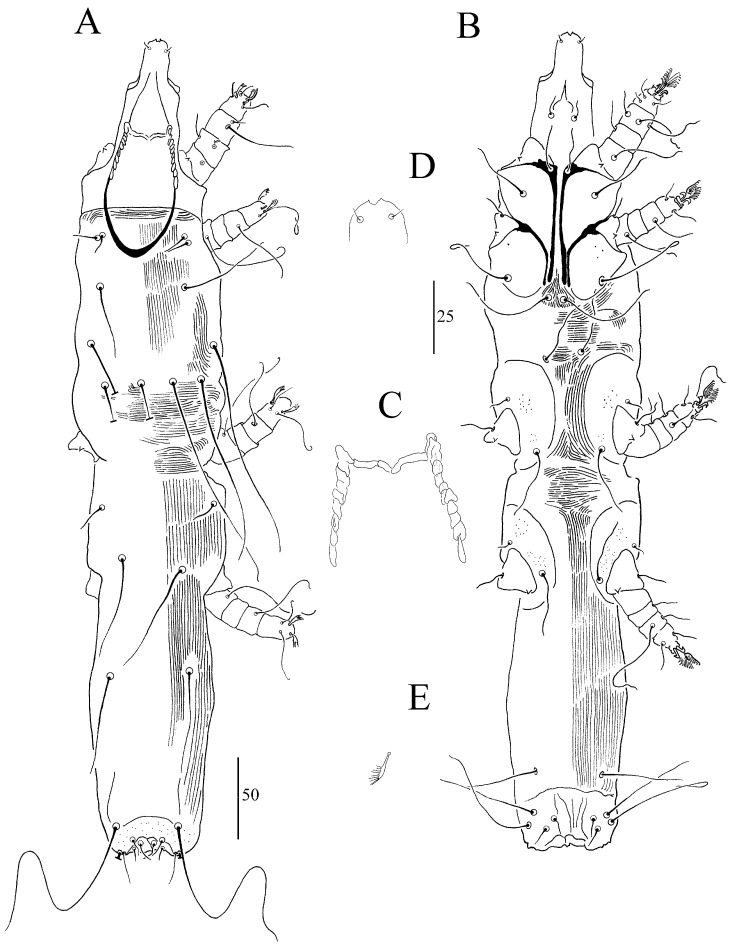
(**A**–**E**). *Syringophiloidus campephilus* sp. n., female: (**A**)—dorsal view, (**B**)—ventral view, (**C**)—peritremes, (**D**)—hypostomal apex, (**E**)—fan-like setae *p’* of leg III. Scale bars: (**A**,**B**) = 50 µm; (**C**–**E**) = 25 µm.

Female (holotype and 7 paratypes; range in parentheses). Total body length 665 (650–655). *Gnathosoma*. Infracapitulum apunctate or sparsely punctate. Each medial and lateral branch of peritremes with 2–3 and 8–10 chambers, respectively (Figure 5C). Stylophore apunctate, 150 (150) long. *Idiosoma.* Propodonotal shield weakly sclerotized punctate around bases of setae *ve*. Length ratio of setae *vi*:*ve*:*si* 1:1–1.6:1–1.6. Hysteronotal shield weakly sclerotized and apunctate, fused to pygidial shield. Pygidial shield sparsely punctate. Setae *f1* and *h1* subequal in length. Length ratio of setae *ag1*:*ag2*:*ag3* 1:1.1–1.9:1.5–1.9. *Legs.* Coxal fields I–II sparsely punctate, III–IV punctate. Setae *3c* 3.3–5 times longer than *3b*. Fan-like setae *p’* and *p”* of legs III–IV with 6–7 tines (Figure 5E). Setae *tc”* 1.4–1.6 times longer than *tc’*. *Lengths of setae*: *vi* 15 (15); *ve* 25 (20–25); *si* 25 (15–20); *c2* 145 (145–170); *se* 180 (195); *c1* 180 (170–195); *d2* 15 (10–15); *d1* 70 (65–80); *e2* (70–90); *f1* 15 (10–20); *f2* 205 (215–235); *h1* 15 (15–20); *h2* 285; *ag1* 65 (55–60); *ag2* 70 (75–105); *ag3* 100 (105); *g1*, *g2* 15 (15); *ps1* 15 (15); *tc’* 25 (20–25); *tc”* 35 (30–40); *l’RIII* 25 (20–25); *l’RIV* 25 (15–20); *3b* 10 (10–15); *3c* 50 (35–50); *4b* 10 (10–15); *4c* 50 (35–50).

Host and Distribution

Birds of the family Picidae: guayaquil woodpecker *Campephilus gayaquilensis* (Lesson) from Peru.

Type Material

Female holotype and 7 female paratypes from the quill of the guayaquil woodpecker *Campephilus gayaquilensis* (Lesson) (Piciformes: Picidae) (USNM 643881), PERU, Tumbes, El Caucho Biological Station, 3 49 25 S, 80 15 37 W, 9 Jun 2009, bird coll. Vargas, W.; mites sampled by Glowska E.; vouchers and DNA codes: EG964, EG971; DNA barcode GenBank accession numbers as specified in Table 1.

Type Material Deposition

Female holotype (USNMENT acc. number: USNMENT01967011) and 3 paratypes (USNMENT01967012–USNMENT01967014) are deposited in the USNM and 4 female paratypes in the AMU (EG23-0628-004.01–04).

Differential Diagnosis

*Syringophiloidus campephilus* sp. n. is morphologically most similar to *S. atlapetes* sp. n. described from the white-headed brushfinch *Atlapetes albiceps* (Taczanowski) (Passeriformes: Passerellidae) from Peru. Females of both species have a similar number of peritremal chambers, punctate pygidial shields and fan-like setae with 6–7 tines. Females of *S. campephilus* sp. n. differ from *S. atlapetes* sp. n by the apunctate stylophore (vs. punctate in *S. atlapetes*) and lengths of setae *si* 15–25 (vs. 65–75), *d2* 10–15 (vs. 165–175), *d1* 65–80 (vs. 145), *e2* 70–90 (vs. 125–170), *ag1* 55–65 (vs. 125) and *ag3* 100–105 (vs. 160). The genetic distance between these species equals 17.3% of K2P (and 15.1% of distance *p*). *S. campephilus* sp. n. is also very similar to *S. dendrocittae* Fain, Bochkov and Mironov, 2000 described from the rufous treepie *Dendrocitta rufa* Baker (Passeriformes: Corvidae) from East Asia [32]. Females of both species have a similar number of peritremal chambers and fan-like setae *p’* and *p”* of legs III–IV with 6–8 tines. Females of *S. campephilus* sp. n. differ from *S. dendrocittae* by the lengths of the setae *vi* 15 (vs. 24), *ve* 20–25 (vs. 45), *d2* 10–15 (vs. 94), *d1* 65–80 (vs. 157), *e2* 70–90 (vs. 132), *ag1* 55–65 (vs. 128–157), *ag2* 70–105 (vs. 135), *ag3* (vs. 166), *g1,2* 15 (vs. 33), *ps1,2* 15 (vs. 27).

Etymology

The name is taken from the generic name of the host and is a noun in apposition.

##### *Syringophiloidus mahali* sp. n. (Figure 6A–E)

**Figure 6:**
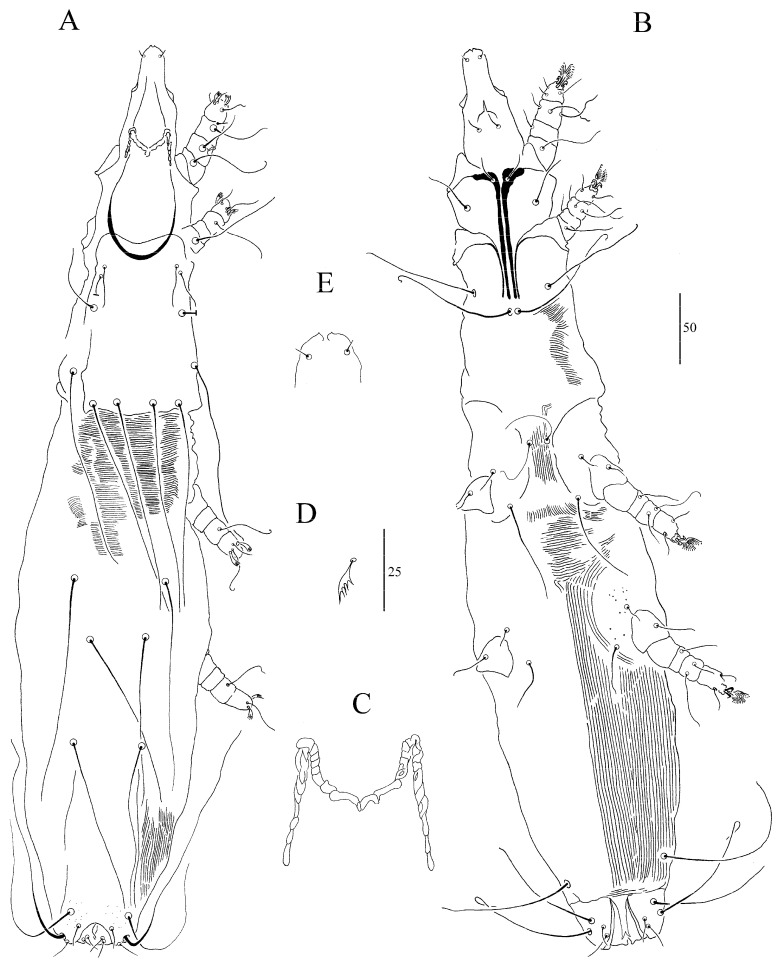
(**A**–**E**). *Syringophiloidus mahali* sp. n., female: (**A**) dorsal view, (**B**) ventral view, (**C**) peritremes, (**D**) hypostomal apex, and (**E**) fan-like setae *p’* of leg III. Scale bars: (**A**,**B**) = 50 µm; (**C**–**E**) = 25 µm.

In terms of females (a holotype and six paratypes; range in parentheses), the total body length is 785 (715–785). For *Gnathosoma*, the infracapitulum is sparsely punctate. Each medial and lateral branch of peritremes has 5–6 and 10–11 chambers, respectively (Figure 6C). The stylophore is apunctate and 185 (170–180) long. For *Idiosoma*, the propodonotal shield is anteriorly concave and apunctate. The length ratio of setae *vi*:*ve*:*si* is 1:1.1–1.3:1.1–1.8. The hysteronotal shield is apunctate and fused to the pygidial shield. The pygidial shield is distinctly sclerotized and punctate in the area bearing bases of setae *f1* and *f2*. Setae *h1* is 1.2–1.3 times longer than *f1*. The length ratio of setae *ag1*:*ag2*:*ag3* is 1–1.2:1–1.2:1.1–1.3. Setae *ps2* is 1.3–1.4 longer than *ps1*. Setae *g1* and *g2* are subequal in length. For *Legs*, Coxal fields I–IV are sparsely punctate. Setae *3c* is 3–3.6 times longer than *3b*. Fan-like setae *p’* and *p”* of legs III–IV have six to seven tines (Figure 6E). Setae *tc”* is 1.5–2 times longer than *tc’*. Lengths of setae are as follows: *vi* 30 (25–30); *ve* 40 (30–35); *si* 55 (35–45); *c2* 155 (135–160); *se* 185 (175–195); *c1* 170 (180); *d2* 150 (155–180); *d1* 130 (145–155); *e2* 150 (155–170); *f1* 20 (15–20); *f2* 120 (130–150); *h1* 25 (20–25); *h2* 285 (290–295); *ag1* 150 (110–130); *ag2* 120 (115–130); *ag3* 130 (145–150); *g1*, *g2* 25 (20); *ps1* 15 (10–15); *ps2* 20 (15–20); *tc’* 35 (20–30); *tc”* 55 (40–45); *l’RIII* 30 (30–35); *l’RIV* 30 (25); *3b* 25 (25); *3c* 80 (75–90); *4b* 25 (20–25); *4c* 90 (85–65).

Host and Distribution

Birds of the family Ploceidae: the white-browed sparrow-weaver, *Plocepasser mahali* Smith from Namibia.

Type Material

The type material consisted of a female holotype and six female paratypes from the quill of the white-browed sparrow-weaver, *Plocepasser mahali* Smith (Passeriformes: Ploceidae) (USNM 642639), Namibia, Hardap, Aukens, 25 09 03 S, 16 32 00 E, 29 Aug 2009, coll. Mughongora, V. K. Mites were sampled by Glowska E.; vouchers and DNA codes are as follows: KR047-048 and KR052. DNA barcode GenBank accession numbers are specified in Table 1.

Type Material Deposition

A female holotype (USNMENT acc. number: USNMENT01967015) and three paratypes (USNMENT01967016–USNMENT01967018) are deposited in the USNM; three female paratypes are deposited in the AMU (EG23-0628-005.01–03).

Differential Diagnosis

*Syringophiloidus mahali* sp. n. is morphologically most similar to *S. picidus* Skoracki, Klimovičová, Muchai and Hromada, 2014, described from the cardinal woodpecker *Dendropicos fuscescens* and recorded in Kenya, Tanzania and Uganda [31]. Females of both species have a similar number of peritremal chambers, a hysteronotal shield fused to the pygidial shield, a punctate pygidial shield in the posterior part, and punctate Coxal fields I–IV. Females of *S. mahali* sp. n. differ from those of *S. picidus* in terms of the length of the stylophore, which is 170–185 (vs. 155–170 in *S. picidus*), the setae *ps2*, which is 1.3–1.4 longer than *ps1* (vs. *ps1*,*2* subequal in length), and the lengths of setae *si*, which are 35–55 (vs. 60–80), of *c1*, which are 170–180 (vs. 210–215), of *f2*, which are120–150 (vs. 150–180), and of *h2*, which are 285–295 (vs. 315–395). *S. mahali* sp. n. is also very similar to *S. philomelosus* Skoracki, 2011, described from the song thrush *Turdus philomelos* Brehm (Passeriformes: Turdidae) from Jordan [22]. Females of both species have a similar number of chambers in the lateral branches, fan-like setae *p’* and *p”* of legs III–IV with six to seven tines and lengths of most setae. Females of *S. mahali* sp. n. differ from those of *S. philomelosus* in terms of the number of chambers of the medial branch of peritremes (5–6 and 8–10 in *S. mahali* sp. n. and *S. philomelosus*, respectively), fused hysteronotal and pygidial shields (vs. not fused) and lengths of setae *c1* of 170–180 (vs. 220–225), *c2* of 135–160 (vs. 175–180), *f1* of 15–20 (vs. 30), *f2* of 120–150 (vs. 190–200), *h2* of 285–295 (vs. 345), *tc’* of 20–35 (vs. 40–45), and *tc”* of 40–55 (vs. 65).

Etymology

The name is taken from the generic name of the host and is a noun in apposition.

##### *Syringophiloidus paludicolae* sp. n. (Figure 7A–E)

**Figure 7:**
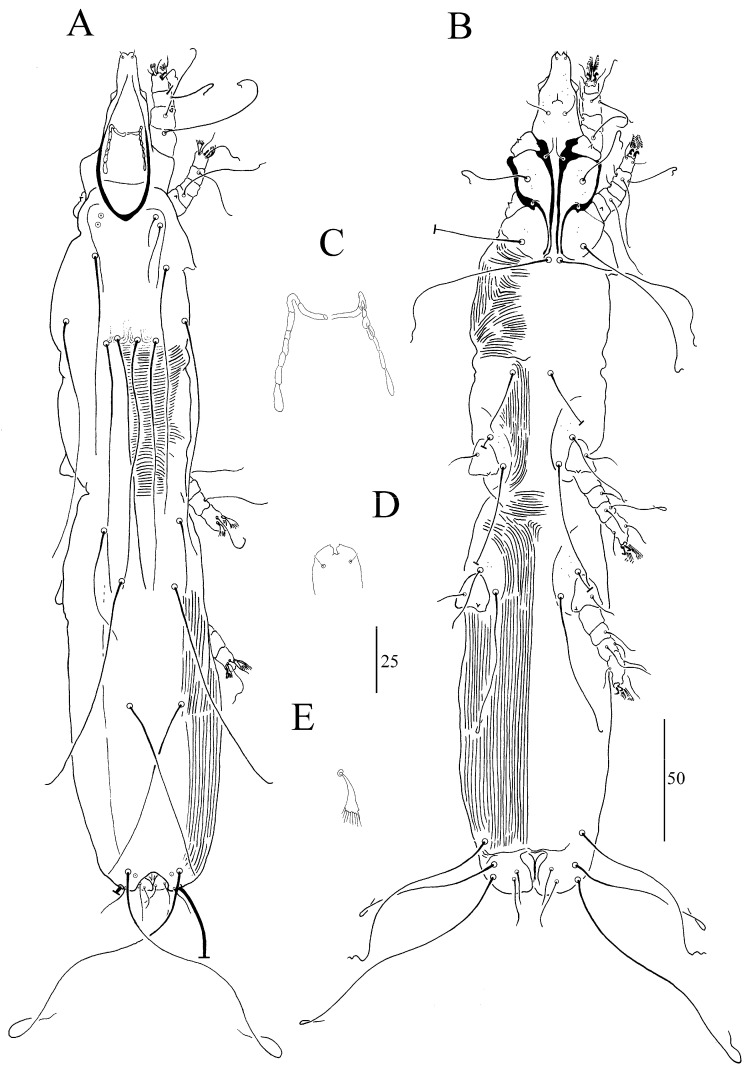
(**A**–**E**). *Syringophiloidus paludicolae* sp. n., female: (**A**) dorsal view, (**B**) ventral view, (**C**) peritremes, (**D**) hypostomal apex, and (**E**) fan-like setae *p’* of leg III. Scale bars: (**A**,**B**) = 50 µm; (**C**–**E**) = 25 µm.

In terms of females (a holotype and seven paratypes; range in parentheses), the total body length is 800 (770–835). For *Gnathosoma*, the infracapitulum is sparsely punctate. Each medial and lateral branch of peritremes has one to two and seven to eight chambers, respectively (borders are poorly marked) (Figure 7C). The stylophore is apunctate and has a length of 160 (150–155). For *Idiosoma*, the propodonotal shield is weakly sclerotized and apunctate. The length ratio of setae *vi*:*ve*:*si* is 1:1.5–1.6:5.3–6. The hysteronotal shield is weakly sclerotized (striation is clearly visible on the entire surface) and apunctate. The pygidial shield is distinctly sclerotized and sparsely punctate in the area bearing bases of setae *f1* and *f2*, while the upper part is weakly sclerotized. Setae *h1* is 1.1 times longer than *f1*. The length ratio of setae *ag1:ag2:ag3* ois 1.1–1.3:1:1.1–1.6. For *Legs,* Coxal fields I–IV are sparsely punctate. Setae *3c* is 2.5–2.9 times longer than *3b*. Fan-like setae *p’* and *p”* of legs III–IV have seven to eight tines (Figure 7E). Setae *tc”* is 1.6–2.4 times longer than *tc’*. Lengths of setae are as follows: *vi* 30 (30); *ve* 50 (45); *si* (160–180); *c2* 230 (220–250); *se* 270 (255–265); *c1* 270 (270–285); *d2* 115 (105); *d1* 195 (170–180); *e2* 180 (160–180); *f1* 35 (35–40); *f2* (305–330); *h1* 40 (40–45); *h2* 420 (400–410); *ag1* 175 (170–195); *ag2* 155 (130–175); *ag3* 205 (195–230); *g1*, *g2* 40 (40–50); *ps1* 20 (25); *ps2* 35 (40); *tc’* 35 (25–35); *tc”* 50 (55–60); *l’RIII* 55 (40–55); *l’RIV* 30 (25–35); *3b* 40 (40–50); *3c* 110 (115–125); *4b* 35 (30–45); *4c* 120 (95–135).

Male: not found.

Host and Distribution

Birds of the family Hirundinidae: the plain martin, *Riparia paludicola* (Vieiilot) from Namibia.

Type Material

The type material consists of a female holotype and seven female paratypes from the quill of the plain martin, *Riparia paludicola* (Vieiilot) (Passeriformes: Hirundinidae) (USNM 642532), NAMIBIA, Karas, Sandfontein near Orange River, 28 51 45 S, 18 33 08 E, 17 Aug 2009, bird specimen coll. Gebhard C. A., mites are sampled by Glowska E.; vouchers and DNA codes are as follows: KR055-056. DNA barcode GenBank accession numbers are specified in Table 1.

Type Material Deposition

A female holotype (USNMENT acc. number: USNMENT01967019) and three paratypes (USNMENT01967020–USNMENT01967022) are deposited in the USNM, and four female paratypes are deposited in the AMU (EG23-0628-002.01–04).

Differential Diagnosis

*Syringophiloidus paludicolae* sp. n. is morphologically most similar to *S. tarnii* Skoracki and Sikora, 2002, described from the huet huet *Pteroptochos tarni* (King) (Passeriformes: Rhinocryptidae) from Argentina [33]. Females of both species have a punctate infracapitulum, a weakly sclerotized and apunctate hysteronotal shield, fan-like setae *p’* and *p”* of legs III–IV with six to eight tines, and similar lengths of most setae. Females of *S. paludicolae* sp. n. differ from those of *S. tarnii* in terms of the number of peritremal chambers, i.e., one to two and seven to eight in medial and lateral branches (vs. three to four and nine), and lengths of setae *c2* of 220–250 (vs. 155–205), *se* of 255–270 (vs. 165–225), *c1* of 270–285 (vs. 190–240), *d1* of 170–195 (vs. 125–145), *e2* of 160–180 (vs. 115–155), and *f2* of 305–330 (vs. 250–280). *S. paludicolae* sp. n. is also very similar to *S. ripariae* sp. n., described from the sand martin, *Riparia riparia* (L.) (Passeriformes: Hirundinidae) from Poland (p.p.). Females of both species are similar in length and weakly sclerotized, have a similar number of peritremal chambers, and setae *g1* and *g2* that are subequal in length. Females of *S. paludicola* sp. n. differ from those of *S. ripariae* sp. n. in terms of the lengths of setae *ve* of 45–50 (vs. 35 in *S. paludicolae*), *d2* of 105–115 (vs. 180), *h1* of 40–45 (vs. 30), and *ps2* of 35–40 (vs. 25). The genetic distance between these species is 15.3% of K2P (and 13.7 of distance *p*).

Etymology

The name is taken from the specific name of the host and is a noun in the genitive case.

##### *Syringophiloidus ripariae* sp. n.

Female (holotype). Total body length 820. *Gnathosoma*. Infracapitulum punctate. Each medial and lateral branch of peritremes with 2 and 7 chambers, respectively. Stylophore apunctate, 180 long. *Idiosoma.* Propodonotal shield weakly sclerotized and apunctate. Length ratio of setae *vi*:*ve* 1:1.4. Hysteronotal shield weakly sclerotized (striation visible on the entire surface) and apunctate. Pygidial shield sparsely punctate, distinctly sclerotized in the area bearing bases of setae *f1* and *f2*, upper part weakly sclerotized. Setae *f1* 1.1 longer than *h1*. Length ratio of setae *ag1*:*ag2*:*ag3* 1.3–1:1.7 *Legs.* Coxal fields I sparsely punctate, III–IV apunctate. Setae *3c* 1.4 times longer than *3b*. Fan-like setae *p’* and *p”* of legs III–IV with 6 tines. *Lengths of setae*: *vi* 25; *ve* 35; *c2* 205; *se* 260; *c1* 275; *d2* 180; *d1* 180; *e2* 180; *f1* 32; *f2* 300; *h1* 28; *h2* 355; *ag1* 170; *ag2* 130; *ag3* 220; 40; *ps1* 20, *ps2* 25; *l’RIII* 45; *l’RIV* 40; *3b* 55; *3c* 80; *4b* 35; *4c* 100.

Male: not found.

Host and Distribution

Birds of the family Hirundinidae: the sand martin *Riparia riparia* (L.) from Poland.

Type Material

The type material was a female holotype from the quill of the sand martin, *Riparia riparia* (L.) (Passeriformes: Hirundinidae), Poznań, POLAND, 52.4672007265976, 16.924954974622207, April 2009, coll. Glowska E.; the voucher and DNA code are as follows: EG079 (holotype). DNA barcode GenBank accession numbers are specified in Table 1.

Type Material Deposition

The holotype was accidentally crushed after species diagnosis was carried out and before the specimen was drawn.

Differential Diagnosis

*Syringophiloidus ripariae* sp. n. is morphologically most similar to *S. tarnii* Skoracki and Sikora, 2002, described from the huet huet *Pteroptochos tarnii* (Passeriformes: Rhinocryptidae) from Argentina [33]. Females of both species have a punctate infracapitulum, a weakly sclerotized and apunctate hysteronotal shield, fan-like setae *p’* and *p”* of legs III–IV with six tines, and similar lengths of most setae. Females of *S. ripariae* sp. n. differ from those of *S. tarnii* in terms of the number of peritremal chambers, i.e., two and seven in the medial and lateral branches (vs. 3–4 and 9), and lengths of setae *se* of 260 (vs. 165–225), *c1* of 275 (vs. 190–240), *d2* of 180 (vs. 130), *d1* of 180 (vs. 125–145), *e2* of 180 (vs. 115–155), and *ag3* of 220 (vs. 145–185). *Syringophiloidus ripariae* sp. n. is also very similar to *S. paludicolae* sp. n. described from the plain martin, *Riparia paludicola* (Vieillot), from Namibia (p.p.). See the *S. paludicolae* sp. n. differential diagnosis that is given above.

Etymology

The name is taken from the specific name of the host and is a noun in the genitive case.

#### 3.3.2. Other Species

##### *Syringophiloidus amazilia* Skoracki, 2017

*Syringophiloidus amazilia* Skoracki, 2017: 181.

Type host: *Chlorestes candida* (Bourcier and Mulsant) (Apodiformes: Trochilidae). Type locality: Mexico.

Host and Distribution

Birds of the family Trochilidae: the white-bellied emerald, *Amazilia candida* (Bourcier and Mulsant), from Mexico [34].

Material Examined

The material examined included one female from the quill of the white-bellied emerald, *Amazilia candida* (Bourcier and Mulsant) (Apodiformes: Trochilidae), Mexico, Veracruz, Los Tuxtlas, 9 May 2008, coll. S.V. Mironov (SVM 08-0509-8/4). Specimen vouchers and DNA codes are as follows: EG880. DNA barcode GenBank accession nos. are given in Table 1.

Material Deposition

Material deposited in the AMU (EG23-0628-008.01).

Remark

Our results revealed that *S. stawarczyki* and *S. amazilia* are conspecific, and as a consequence, *S. amazilia* could be treated as a junior synonym of *S. stawarczyki*. Although our results are precise, they are based on a relatively small sample. This is due to the limited availability of the mite material. For this reason, we do not formally synonymize these species, but only formulate a premise for further systematic research on the populations covering a more significant number of individuals.

##### *Syringophiloidus glandarii* (Fritsch,1958)

*Syringophilus minor glandarii* Fritsch, 1958: 235.

Syringophilus glandarii as incertae sedis Kethley 1970: 65.

*Syringophiloidus glandarii* Bochkov and Mironov 1998: 14.

Type host: *Garrulus glandarius* L. (Passeriformes: Corviidae)

Type locality: Germany.

Host and Distribution

Birds of the family Corvidae: the eurasian jay, *Garrulus glandarius* (L)., eurasian magpie, *Pica pica* (L.), eurasian jackdaw, *Corvus monedula* L., rook *Corvus frugilegus* L. [22], American crow, *Corvus brachyrhynchos* Brehm, steller’s jay, *Cyanocitta stelleri* (Gmelin) [35], and hooded crow, *Corvus corone* L. (p.p.) from Germany [36], Russia, Kazakhstan, Japan [22], USA [35], and Poland (p.p.).

Material Examined

Four females from the quill of the hooded crow *Corvus corone cornix* L. (Passeriformes: Corvidae) were used, and the material obtained from dead birds (due to probable collisions with a window) was found on the AMU campus, Poznań, Poland (8 May 2009), coll, Glowska E. Specimen vouchers and DNA codes are as follows: EG519 and EG522. DNA barcode GenBank accession nos. are given in Table 1.

Material Deposition

Material is deposited in the AMU (EG23-0628-13.01–04).

##### *Syringophiloidus parapresentalis* Skoracki, 2011

*Syringophiloidus parapresentalis* Skoracki, 2011: 63.

Type host: *Turdus merula* L. (Passeriformes: Turdidae)

Type locality: Poland.

Host Range and Distribution

Birds of the family Turdidae: the Eurasian blackbird, *Turdus merula* L., fieldfare, *T. pilaris* L., black-throated thrush, *T. atrogularis* Jarocki [22], and redwing *T. iliacus* L. ([22], p.p.) from Slovakia, Kazakhstan, Russia and Jordan [22], and Poland ([22], p.p.).

Material Examined

Five females from the quill of the redwing *Turdus iliacus* L. (Passeriformes: Turdidae) made up the material examined, and the material was obtained from dead birds (due probable collision with glass) found on the AMU campus, Poznań, Poland (16 July 2009), coll, Glowska E. Specimen vouchers and DNA codes are as follows: EG019, EG061-063. DNA barcode GenBank accession nos. are given in Table 1.

Material Deposition

Material is deposited in the AMU (EG23-0628-14.01–05).

##### *Syringophiloidus picidus* Skoracki, Klimovičová, Muchai and Hromada, 2014

*Syringophiloidus picidus* Skoracki, Klimovičová, Muchai and Hromada, 2014: 184.

Type host: *Dendropicos fuscescens* (Vieillot) (Piciformes: Picidae)

Type locality: Kenya.

Host and Distribution

Birds of the family Picidae: the cardinal woodpecker, *Dendropicos fuscescens* (Vieillot), from Kenya, Tanzania, Uganda [31], Namibia (p.p).

Material Examined

Two females from the quill of the cardinal woodpecker, *Dendropicos fuscescens* (Vieillot) (Piciformes: Picidae), NAMIBIA, 14 August 2009, Karas, Oas, 27 29 43 S, 19 13 14 E, bird coll. Gebhard C. A., mite coll. Glowska E (USNM 642511), were examined. Specimen vouchers and DNA codes are as follows: KR031; KR033. DNA barcode GenBank accession nos. are given in Table 1.

Material Deposition

One female deposited in the USNM (USNMENT acc. number: USNMENT01967023) and one female in the AMU (EG23-0628-009.01).

##### *Syringophiloidus plocei* Glowska, Broda, Gebhard and Dabert, 2016

*Syringophiloidus plocei* Glowska, Broda, Gebhard and Dabert, 2016: 563.

Type host: *Ploceus cucullatus* (St. Muller) (Passeriformes: Ploceidae).

Type locality: Gabon.

Host and Distribution

Birds of the family Ploceidae: the village weaver, *Ploceus cucullatus* (Müller), and Vieillot’s black weaver, *Ploceus nigerrimus* Vieillot [15].

Material Examined

Four females from the quill of the village weaver, *Ploceus cucullatus* (St. Muller) (Passeriformes: Ploceidae) GABON, Ogooue Maritime Province, Gamba Complex of Protected Areas, near the mouth of Nyanga River, 22 October 2009, bird host coll. C.A. Gebhard, were sampled; mites were sampled by E. Glowska (September 2014) (USNM 642906). Four females from the Vieillot’s black weaver, *Ploceus nigerrimus* Vieillot (Ploceidae), GABON, Estuaire Province, Cap Esterias, National Forestry School (ENEF), 3 November 2009, bird host coll. C.A. Gebhard, were also sampled; mites were sampled by E. Glowska (USNM 642955). Specimen vouchers and DNA codes are as follows: GE038-039; GE041-042. DNA barcode GenBank accession nos. are given in Table 1.

Material Deposition

Two females from each species (the village weaver and the Vieillot’s black weaver) are deposited in the USNM (USNMENT acc. number: USNMENT01967024–USNMENT01967027) and in the AMU (EG23-0628-011.01–04).

##### *Syringophiloidus pseudonigritae* Glowska, Dragun-Damian and Dabert, 2012

*Syringophiloidus pseudonigritae* Glowska, Dragun-Damian and Dabert, 2012.

Type host: *Pseudonigrita arnaudi* (Bonaparte) (Passeriformes: Ploceidae).

Type locality: Tanzania.

Host and Distribution

Birds of the family Ploceidae: the grey-headed social weaver, *Pseudonigrita arnaudi* (Bonaparte), from Tanzania (Glowska et al., 2012) [10].

Material Examined

Four females from the quill of the frozen specimen of the grey-headed social weaver, *Pseudonigrita arnaudi* (Bonaparte) (Passeriformes: Ploceidae), were examined; the bird host was initially collected from the wild in Tanzania and imported to Hamburg in 1990 where it was housed in the Biozentrum Grindel and Hamburg Zoological Museum in the University of Hamburg, Germany, coll. E. Glowska, November 2010.

Specimen vouchers and DNA codes are as follows: EG545-547. DNA barcode GenBank accession nos. are given in Table 1.

Material Deposition

Material is deposited in the AMU (EG23-0628-12.01–04).

##### *Syringophiloidus sporophila* Skoracki, 2017

*Syringophiloidus sporophila* Skoracki, 2017: 184.

Type host: *Sporophila torqueola* (Bonaparte) (Passeriformes: Thraupidae). Type locality: Mexico.

Host and Distribution

Birds of the family Thraupidae: the cinnamon-rumped seedeater, *Sporophila torqueola* (Bonaparte), from Mexico (Skoracki 2017) [34].

Material Examined

Ten females from the quill of the cinnamon-rumped seedeater, *Sporophila torqueola* (Bonaparte) (Passeriformes: Thraupidae), Mexico, Veracruz, Los Tuxtlas, 6 May 2008, coll. S.V. Mironov (SVM 08-0506-1/4), were used. Specimen vouchers and DNA codes are as follows: EG362–366, and EG688–692. DNA barcode GenBank accession nos. are given in Table 1.

Material Deposition

Material deposited in the AMU (EG23-0628-006.01–10).

##### *Syringophiloidus stawarczyki* Skoracki, 2004

*Syringophiloidus stawarczyki* Skoracki, 2004: 291.

Type host: *Euphonia cyanocephala* (Vieillot) (Passeriformes: Emberizidae). Type locality: Brazil.

Host and Distribution

Birds of the families Emberizidae and Thraupidae: the golden-rumped euphonia, *Euphonia cyanocephala* (Vieillot) (type host), white-lined tanager, *Tachyphonus rufus* (Boddaert) [29], and blue dacnis, *Dacnis cayana* (L.) [30].

Material Examined

Two females from the quill of the blue dacnis *Dacnis cayana* (Linnaeus) (Passeriformes: Thraupidae), Brazil, Minas Gerais, Nova Lima, APP do Condomínio Miguelão, 20°07′17.2″ S 43°58′03.1″ W, 8 September 2010, coll. S.V. Mironov, F.A. Hernandes & M.P. Valim (field no. SVM 10-0908-1–2), were examined. Specimen vouchers and DNA codes are as follows: EG854–855. DNA barcode GenBank accession nos. are given in Table 1.

Material Deposition

Materials are deposited in the AMU (EG23-0628-007.01–02).

## 4. Discussion

Both topologies of the phylogenetic trees and genetic distances revealed thirteen strongly supported monophyletic lineages which are in most cases in accordance with the morphological identifications. The only exception is *S. amazilia*, which very close to the *S. stawarczyki* clade and most likely represents a population of this species. This result is further supported by the genetic distance between the two lineages (1.4% and 1.5% of distances *p* and K2P, respectively), which is lower than that between bihost *S. plocei* populations (2.1%) and comparable to the previously reported intraspecific values within other quill mites [8,16]. Also, a morphological analysis of the type material of both species showed that they are almost indistinguishable and share most diagnostic characteristics (both qualitative and quantitative). The differences between the alleged “species” are very subtle and manifest only in the length of setae *d2* (135–170 in females of *S. amazilia* vs. 115–125 in *S stawarczyki*), *f2* (175 vs. 220), *ag1* (105–120 vs. 130–135), and *ag2* (100–110 vs. 125–135). It is very likely that the differences are caused by the fact that both species were described based on a few specimens only (seven and three females of *S. amazilia* and *S. stawarczyki*, respectively) [29,34]. This is a common practice when researchers work with hard-to-reach and low-prevalence material. It seems, however, that more individuals’ availability would fill the metric data gap between *S. amazilia* and *S. stawarczyki* and show the continuity of the divergent characters. The presence of the same mite species on two phylogenetically distant hosts (representatives of different orders, i.e., Apodiformes and Passeriformes) can be explained by horizontal transfer since the ranges of both hosts overlap in Central America. At the moment, we do not have sufficient data to point the direction of the transfer. To carry this out, more individuals representing more populations of both hosts should be analyzed The cases of the host switching of quill mites have already been reported and our result supports the earlier assumption that this phenomenon is not incidental but rather one of the possible scenarios for the dispersion and evolution of this group of parasites [16,37].

In all other cases, the analysis of molecular data (NJ and genetic distances) confirmed the morphological separateness of previously known and newly described species. The intraspecific distances of all tested taxa were not higher than 0.9% and were comparable to the interpopulation values, i.e., 1.5% between *S. plocei* from the vieillot’s black weaver and village weaver. All these values are similar to those previously observed in other stenoxenous quill mites (0.0–2.3) [8,14]. Also, interspecific diversity, which ranged from 5.9% to 19.2% and 6.3–22.4% based on distance *p* and K2P, respectively (Table 2), is comparable to that among the species in other previously barcoded syringophilid genera [16].

Although all putative species (except *S. amazilia*) are highly supported with bootstrap values (100%), the relationships between them have not been fully resolved and only faintly indicate that both the host phylogeny and distributions may influence the phylogenetic structure of mites. For example, *S. ripariae* sp. n. from Poland and *S. paludicolae* sp. n. from Namibia were both recorded from hirundinid birds. Their populations show clear intraspecific integrity as well as species separateness measured via genetic distance (16.3% and 14.5% of K2P and *p*, respectively). Even though both species come from geographically distant locations, they form a sister group on the phylogenetic tree. This may suggest a parallel evolution of mites with avian hosts. Another example of a co-phylogenetic relationship is shown by *S. plocei* found on two ploceid species in Namibia. This clade forms a sister group with *S. pseudonigritae*, a parasite of another ploceid bird, the grey-headed social weaver in Tanzania. This result confirmed our earlier observations for these taxa (Glowska et al. 2016) [15]. Another factor that may shape the phylogenetic structure of mites is geographical distribution. Two species, *S. glandarii* and *S. parapresentalis*, form a statistically well-supported sister group. Although they were obtained from birds from different families (Corvidae and Turdidae, respectively), they have a common location (Poland). Analogously, two species parasitize separate bird orders, *S. calamonastes* sp. n. and *S. picidus* form the “Namibian cluster”. The same can be observed with the clearly distinct clade represented by mites from Mexico and South America (*S. atlapetes*; *S. Stawarczyki-S. amazilia*).

In this work, we used morphological and barcode data to estimate the diversity and genetic variability of fifteen populations of the genus *Syringophiloidus*. In most cases, both sources of information were consistent. The only exception was *S. amazilia*, which seems to be a population of *S. stawarczyki* and formally should be treated as its junior synonym. The further findings of our study are six now-to-science species, described herein. We indicate that both host phylogeny and distribution can drive the evolution of quill mites. However, we treat our results as a starting point for further in-depth research on these issues. Our results increase the knowledge about mite diversity and demonstrate the usefulness of the parallel use of morphological and molecular methods in solving systematic puzzles in this group of parasites.

## 5. Conclusions

Even though there has been progress in understanding quill mite systematics, little is known about their global diversity and host associations. This is mainly due to the weakly informative morphology and relatively few diagnostic characters. To address this challenge, a combination of classical morphology and DNA barcodes is used to increase the efficiency of species identification. This approach has been proven to be a reliable tool for this purpose, regardless of sex or developmental stage. It is also helpful for estimating genetic diversity and host specificity issues or revealing phenomena resulting from the incorrect interpretation of morphological characters, such as phenotypic plasticity, polymorphisms, or cryptic species.

Accurate species diagnosis is essential for further research, particularly in understanding quill mites’ epidemiological importance. Recent reports suggest that mites host unique phylogenetic lineages of bacteria, such as *Wolbachia* and *Spiroplasma*. Additionally, they are believed to spread diseases by ingesting food (sucking the host’s bodily fluids), although their epidemiological significance has not yet been well studied. Our findings contribute to knowledge about mite diversity and provide a basis for further evolutionary, ecological, and epidemiological investigations.

## Figures and Tables

**Table 1:** Mites and sequences used in the molecular study.

Mite Species	Host Species	Host Order and Family	Location	Specimen and DNA Code	GenBank Access No.
*Syringophiloidus calamonastes* sp. n.	Barred Wren-Warbler *Calamonastes fasciolatus* (Smith)	Passeriformes: Cisticolidae	Namibia	KR043	OR721880
				KR045	OR721881
*S. paludicolae* sp. n.	Plain Martin *Riparia paludicola* (Vieiilot)	Passeriformes: Hirundinidae	Namibia	KR055	OR723490
				KR056	OR723491
*S. ripariae* sp. n.	Bank Swallow *Riparia riparia* (L.)	Passeriformes: Hirundinidae	Poland	EG079	OR723492
				EG080	OR723493
*S. atlapetes* sp. n.	White-headed Brushfinch *Atlapetes albiceps* (Taczanowski)	Passeriformes: Passerellidae	Peru	EG974	OR827223
				EG975	OR827224
				EG976	OR827229
				EG977	OR827227
*S. campephilus* sp. n.	Guayaquil Woodpecker *Campephilus gayaquilensis* (Lesson)	Piciformes: Picidae	Peru	EG964	OR723494
				EG971	OR723495
*S. mahali* sp. n.	White-browed Sparrow-Weaver *Plocepasser mahali* Smith	Passeriformes: Ploceidae	Namibia	KR047	OR827226
				KR048	OR827228
				KR052	OR827225
*S. sporophila* Skoracki, 2017	Cinnamon-rumped Seedeater *Sporophila torqueola* (Bonaparte)	Passeriformes: Thraupidae	Mexico	EG362	OR829593
				EG363	OR829592
				EG364	OR829594
				EG365	OR829596
				EG366	OR829595
				EG688	OR829597
				EG689	OR829598
				EG690	OR829599
				EG691	OR829601
				EG692	OR829602
* *S. stawarczyki* Skoracki, 2004	Blue Dacnis *Dacnis cayana* (Linnaeus)	Passeriformes: Thraupidae	Brazil	EG854	OR829600
				EG855	OR829606
* *S. amazilia* Skoracki, 2017	White-bellied Emerald *Chlorestes candida* (Bourcier and Mulsant)	Caprimulgiformes: Trochilidae	Mexico	EG880	OR829607
*S. picidus* Skoracki, Klimovičová, Muchai and Hromada, 2014	Cardinal Woodpecker *Chloropicus fuscescens* (Vieillot)	Piciformes: Picidae	Namibia	KR031	OR730469
				KR033	OR730471
*S. plocei* Glowska, Broda, Gebhard and Dabert, 2016	Village Weaver *Ploceus cucullatus* (St. Muller)	Passeriformes: Ploceidae	Gabon	GE041	OR829603
				GE042	KU646845.1
	Vieillot’s Black Weaver *Ploceus nigerrimus* (Vieillot)	Passeriformes: Ploceidae	Gabon	GE038	OR829605
				GE039	OR829604
*S. pseudonigritae* Glowska, Dragun-Damian and Dabert, 2012	Gray-headed Social-Weaver *Pseudonigrita arnaudi* (Bonaparte)	Passeriformes: Ploceidae	Tanzania	EG545	OR829610
				EG546	OR829608
				EG547	OR829609
*S. glandarii* (Fritsch, 1958)	Hooded Crow *Corvus corone cornix* L.	Passeriformes: Corvidae	Poland	EG519	OR829611
				EG522	OR829612
*S. parapresentalis* Skoracki, 2011	*Redwing Turdus iliacus* L.	Passeriformes: Turdidae	Poland	EG019	OR829613
				EG061	OR829614
				EG062	OR829615
				EG063	OR829616
*Stibarokris phoeniconaias* Skoracki & OConnor, 2010 outgroup	American flamingo *Phoenicopterus ruber* L.	Galliformes: Phasianidae	Germany	EG642	OR726320

* According to our results, *S. amazilia* and *S. stawarczyki* are conspecific.

**Table 2:** Estimates of evolutionary divergences between COI sequences of *Syringophiloidus* populations based on K2P (and *p*) distances.

Mite Species	Distance *p* (Lower Left) and K2P (Upper Right) (%)																
	Within Groups	Between Groups															
		1.	2.	3.	4.	5.	6.	7.	8.	9.	10.	11.	12.	13.	14.	15.	16.
1. *S. ripariae* sp. n. ex_Bank_Swallow	0.0		16.2	16.0	14.4	16.9	16.3	15.4	15.8	14.2	16.3	17.7	16.0	17.2	17.4	16.2	39.9
2. *S. sporophila* ex Cinnamon-rumped Seedeater	0.2	14.5		17.0	15.6	21.8	15.6	14.2	14.4	16.2	19.6	22.4	15.1	16.6	17.8	17.4	36.8
3. *S. pseudonigritae* ex Gray-headed Social-Weaver	0.0	14.3	15.0		18.7	20.4	15.7	12.6	12.1	18.4	20.2	21.2	19.8	20.2	19.1	18.6	39.4
4. *S. stawarczyki* ex Blue Dacnis	0.0	13.0	14.0	16.3		14.4	6.3	16.2	15.8	1.5	17.8	16.1	15.3	15.5	14.5	13.4	39.5
5. *S. campephilus* sp. n. ex Guayaquil Woodpecker	0.0	15.0	18.7	17.6	12.9		17.3	20.5	21.0	14.6	19.0	19.5	19.0	19.0	19.6	17.5	43.0
6. *S. atlapetes* sp. n. ex White-headed Brushfinch	0.7	14.5	14.0	14.0	5.9	15.1		17.8	17.2	6.3	20.2	18.7	16.4	16.6	15.0	15.2	41.2
7. *S. plocei* ex Village Weaver	0.9	13.9	12.8	11.3	14.5	17.8	15.7		2.1	17.3	18.0	20.1	19.1	19.9	18.3	18.3	37.7
8. *S. plocei* ex Vieillot’s Black Weaver	0.2	14.1	13.0	11.0	14.1	18.1	15.2	2.1		16.6	18.0	19.0	19.5	19.6	17.9	18.3	36.2
9. *S. amazilia* ex White-bellied Emerald	n/a	12.9	14.5	16.1	1.4	13.0	5.9	15.3	14.8		18.3	16.0	16.2	15.1	14.2	14.0	39.5
10. *S. paludicolae* sp. n. ex Plain Martin	0.0	14.5	17.2	17.6	15.8	16.7	17.5	15.9	15.9	16.1		21.9	19.0	21.4	18.5	19.8	39.2
11. *S. mahali* sp. n. ex White-browed Sparrow-Weaver	0.0	15.7	19.2	18.4	14.4	17.1	16.4	17.5	16.7	14.4	18.9		18.6	17.6	19.9	20.6	44.6
12. *S. picidus* ex Cardinal Woodpecker	0.0	14.3	13.6	17.2	13.8	16.7	14.6	16.8	17.0	14.5	16.7	16.2		7.3	21.7	19.5	41.4
13. *S. calamonastes* sp. n. ex Barred Wren-Warbler	0.0	15.2	14.8	17.6	13.9	16.7	14.8	17.4	17.1	13.6	18.5	15.5	6.9		19.9	19.2	38.6
14. *S. parapresentalis* ex Redwing	0.0	15.4	15.8	16.7	13.0	17.0	13.5	16.1	15.8	12.9	16.3	17.5	18.7	17.4		12.1	41.2
15. *S. glandarii* ex Hooded Crow	0.0	14.5	15.4	16.3	12.1	15.4	13.6	16.1	16.1	12.7	17.2	17.9	17.0	16.8	11.1		42.3
16. *Stibarokris phoeniconaias* (outgroup)	-	30.4	28.8	30.1	30.3	32.1	31.1	29.3	28.4	30.3	30.1	33.0	31.2	29.7	31.2	31.7	

## Data Availability

The data presented in this study are available on request from the corresponding author.
